# Determining High-Intensity Sweeteners in White Spirits Using an Ultrahigh Performance Liquid Chromatograph with a Photo-Diode Array Detector and Charged Aerosol Detector

**DOI:** 10.3390/molecules25010040

**Published:** 2019-12-20

**Authors:** Kang Ma, Xiaojia Li, Yiwen Zhang, Fei Liu

**Affiliations:** 1 Division of Chemical Metrology and Analytical Science, National Institute for Metrology of China, Beijing 100013, China; 2 Beijing Key Laboratory of Water Resources and Environmental Engineering, China University of Geosciences (Beijing), Beijing 100083, China; 3 College of Chemistry and Bioengineering, University of Science and Technology Beijing, Beijing 100083, China; lixiaojia_xszx@aliyun.com; 4 Beijing Institute of Metrology, Beijing 100021, China; zhangyw@bjjl.cn

**Keywords:** sweeteners, photo-diode array detector (PDA), charged aerosol detection (CAD), white spirits

## Abstract

In China, white spirit is not only an alcoholic drink but also a cultural symbol. A novel and accurate method for simultaneously determining nine sweeteners (most authorized for use in China) in white spirits by ultrahigh performance liquid chromatography (UHPLC) with a photo-diode array detector (PDA) and charged aerosol detector (CAD) was developed. The sweeteners were acesulfame, alitame, aspartame, dulcin, neotame, neohesperidine dihydrochalcone, saccharin, sodium cyclamate, and sucralose. The sweeteners were separated within 16 min using a BEH C18 column and linear gradient-elution program. The optimized method allowed low concentrations (micrograms per gram) of sweeteners to be simultaneously detected. The CAD gave good linearities (correlation coefficients > 0.9936) for all analytes at concentrations of 0.5 to 50.0 μg/g. The limits of detection were 0.16 to 0.77 μg/g. Acesulfame, dulcin, neohesperidine dihydrochalcone, and saccharin were determined using the PDA detector, which gave correlation coefficients > 0.9994 and limits of detection of 0.16 to 0.22 μg/g. The recoveries were 95.1% to 104.9% and the relative standard deviations were 1.6% to 3.8%. The UHPLC-PDA-CAD method is more convenient and cheaper than LC-MS/MS methods. The method was successfully used in a major project called “Special Action against Counterfeit and Shoddy white spirits” and to monitor risks posed by white spirits in China.

## 1. Introduction

**Figure 1:**
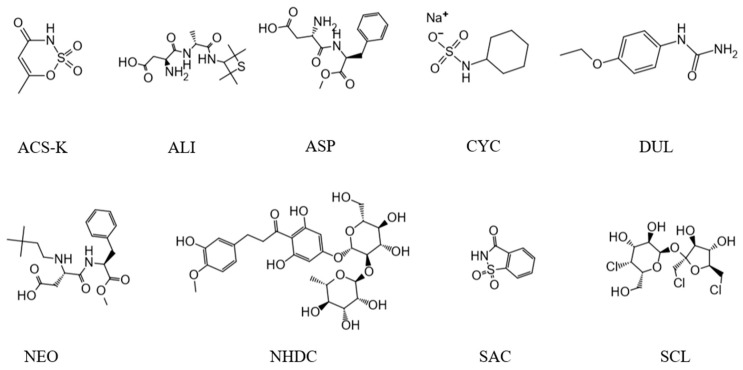
Chemical structures of the sweeteners that were studied.

Humans favor and instinctively desire sweet tastes, resulting in a preference for sweet foodstuffs [1,2]. Legal requirements and consumer pressure to more effectively monitor food safety (for dairy products, sweeteners, and alcoholic products) have recently become important in China. Chinese regulations for sweeteners are published in the national food safety standards for the use of food additives GB 2760 [3]. The maximum concentrations of certain sweeteners in specific types of food are contained in these standards. The standards are constantly revised to keep pace with technological developments in the sweetener field and to ensure that the maximum allowed concentrations of high-potency sweeteners in foods in specific categories are appropriate. Many food products contain sweeteners (singly or in combination), and it is essential that the concentrations of sweeteners in food products are below the maximum concentrations specified in the relevant legislation. The Chinese national food safety standards for wine GB 15037 and GB 2758 [4,5,6] prohibit acesulfame (ACS-K), aspartame (ASP), neotame (NEO), saccharin (SAC), and sodium cyclamate (CYC) (see Figure 1) being added to wine products. According to GB 2760, sucralose were authorized at concentration of 0.65 g/kg in fermented wine. Unfortunately, these sweeteners are often illegally added to various white spirits (which is a subdivision category of fermented wine). The presence of these sweeteners in such products may pose risks to human health, and the sweeteners may cause conditions, such as allergic reactions, bladder cancer, convulsions, hyperpnea, and metabolic acidosis [7,8]. However, some food producers may still add such sweeteners to their products and therefore cause health risks. The sweeteners mentioned above are the most common sweeteners that have been abused in recent years. It is therefore necessary to develop a rapid and accurate method to simultaneously determine the concentrations of these sweeteners in white spirits to ensure that the spirits comply with food safety standards and to assure the public, particularly in China, that spirits being sold met the needs of consumers.

Many “traditional” methods have been developed for determining high-intensity sweeteners in various foodstuffs. These methods are based on various analytical techniques, including high-performance liquid chromatography (HPLC) [9], ion chromatography [10], thin-layer chromatography [11], gas chromatography [12], capillary electrophoresis [13], flow injection analysis [14], electroanalytical techniques [15], nuclear magnetic resonance [16], and spectroscopic techniques [17]. However, most previously developed methods can only be used to analyze one sweetener or simple mixtures of two to four sweeteners. Nowadays, sweeteners are often used as synergistic mixtures to decrease costs and improve the taste of the product, and the maximum permissible amounts of different sweeteners in food vary markedly [18]. It is very important to have an analytical method available for simultaneously determining various sweeteners in various food matrices to allow food quality to be controlled and regulations to be enforced. Wasik et al. [19] developed an HPLC evaporative light-scattering detection method for determining six authorized sweeteners (ACS-K, ASP, CYC, neohesperidine dihydrochalcone (NHDC), SAC, and SCL). Yan et al. [20] used HPLC with evaporative light scattering detection analysis sucralose and related compounds with a better limit of detection (0.5 μg/mL). Koyama et al. [21] developed an LC mass spectrometry (MS) method for simultaneously determining nine sweeteners (ACS-K, ASP, CYC, dulcin (DUL), glycyrrhizic acid, rebaudioside A, SAC, SCL, and stevioside) in various foods. Koyama et al. and Huang [21,22] did not use internal standards (isotopic internal standard) in their methods, and the analytes were detected using the MS instruments in selected ion monitoring mode (SIM) would probably have better relative standard deviations (RSD) if using an isotopic internal standard. LC/tandem MS (MS/MS) is an increasingly popular technique for determining multiple sweeteners in wine and other alcoholic drinks [22,23,24,25,26,27,28,29,30]. Zygler et al. [24] developed an LC-MS/MS method to determine nine European Union-regulated sweeteners in alcoholic beverages. The internal standard was *N*-(2-methylcyclohexyl) sulfamate, and the limit of detection (LOD) was <0.5 μg/g. Chui-Shiang Chang et al. [27] developed an LC-MS/MS method to determine seven sweeteners in alcoholic beverages, and the LOD for each sweetener was 0.1 μg/g. However, LC-MS/MS methods are the most effective, in terms of quantifying the analytes, if isotope-labeled internal standards are used, meaning specially synthesized isotope-labeled sweeteners are required for LC-MS/MS analysis to be effective. LC-MS/MS methods often suffer from matrix effects and require complex sample processing procedures, However, simultaneous analysis of various food sweeteners, including ACS-K, ASP, CYC, NHDC, SAC, and SCL, in wine rapidly and convenience remain an area to be explored. So, LC-MS/MS analyses of sweeteners are more expensive (analysis cost and time) than the ultrahigh performance liquid chromatography (UHPLC) with photo-diode array (PDA) detection and the charged aerosol detection (CAD) method proposed here. Grembecka et al. [31] present a combination of HPLC-CAD-UV/DAD detectors to determine three sweeteners (ACS-K, ASP, and SAC) and two preservatives (citric acid and sodium benzoate) in soft drinks, which was a water soluble matrix. Fermented wine contains sweeteners like sulfonamides, dipeptides, and sucrose derivatives, as well as a complex matrix that is water soluble with chemical families, namely esters, alcohols, terpenic compounds, amino acid, and sulphur compounds, etc. [32]. It is desirable to develop cheap, simple, and fast methods for simultaneous analysis of various synthetic and semi-synthetic high-intensity sweeteners (e.g., ACS-K, alitame (ALI), ASP, CYC, DUL, NEO, NHDC, SAC, and SCL) in wine by HPLC combined with different detectors.

In the presented study, a new method for analyzing multiple sweeteners by UHPLC-PDA-CAD was developed and validated. The method was suitable for analyses to apply the maximum synthetic and semi-synthetic high-intensity sweetener concentrations in ethanol matrix, and make the current standard (food specified in Chinese legislation [3,4,5,6]) more sophisticated. The UHPLC separation method was optimized, and the effects of varying the method parameters on the recoveries, precision, linear range, limits of detection (LODs), and limits of quantification (LOQs) were assessed. The method was used to determine sweeteners in 30 real spirit samples. Finally, the method was successfully used in a major project called “Special Action against Counterfeit and Shoddy white spirits” and to monitor risks posed by white spirits in China.

## 2. Results and Discussion

### 2.1. Optimization of UHPLC Separation

**Figure 2:**
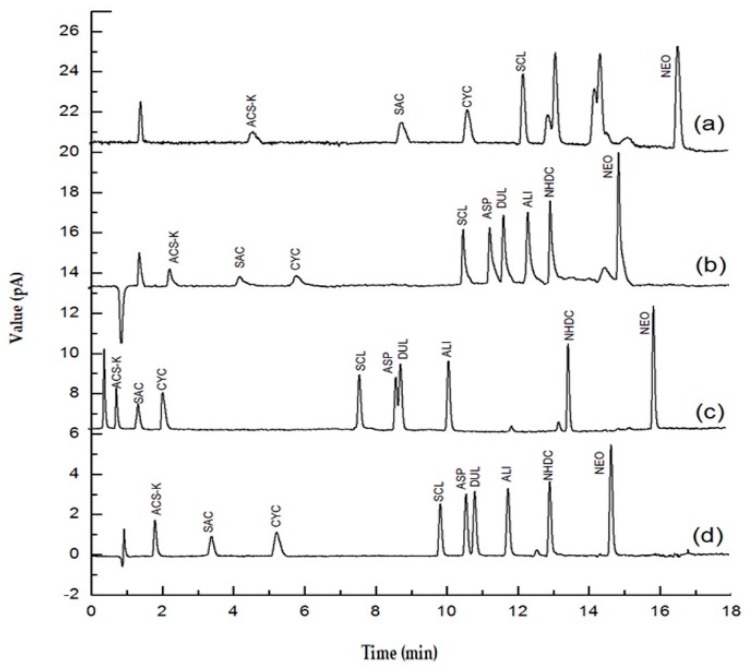
Ultrahigh performance liquid chromatography charged aerosol detector chromatograms for a mixture of nine sweeteners acquired using four different analytical columns, (**a**) a Shim-pack XR-C18 column (3.0 mm × 2.2 μm × 75 mm), (**b**) a Zorbax SB-C18 column (2.1 mm × 1.8 μm × 50 mm), (**c**) an Acquity UPLC BEH C18 column (2.1 mm × 1.7 μm × 50 mm), and (**d**) an Acquity UPLC BEH C18 column (2.1 mm × 1.7 μm × 100 mm).

The first step in developing the new method was selecting an analytical column. A rapid C18 column was selected. This allowed the nine selected sweeteners (ACS-K, ALI, ASP, CYC, DUL, NEO, NHDC, SAC, and SCL) to be eluted within 16 min. Columns of different types from various manufacturers were tested. The columns that were tested were a Shim-pack XR-C18 column (3.0 mm i.d., 75 mm long, 2.2 μm particle size), a Zorbax SB-C18 column (2.1 mm i.d., 50 mm long, 1.8 μm particle size), an Acquity UPLC BEH C18 column (2.1 mm i.d., 50 mm long, 1.7 μm particle size), and an Acquity UPLC BEH C18 column (2.1 mm i.d., 100 mm long, 1.7 μm particle size). As shown in Figure 2, good separation of the nine sweeteners was achieved using every column when the chromatographic conditions were optimized. For more information of the resolution and tailing factor of the nine sweeteners, see the Supplementary Materials in Table S3.

Several parameters (including the mobile phase and the gradient elution parameters) needed to be optimized. Firstly, two complementary detectors, a PDA and CAD, were found to be necessary. The main advantage of using the PDA and CAD in series was that CAD could detect sweeteners regardless of whether they contained chromophores or fluorophores, but the PDA detector could detect ACS-K, DUL, NHDC, and SAC with better sensitivity than that achieved by CAD. Each detector could be used to verify the results of the other detector. Secondly, a buffered mobile phase needed to be selected to give stable retention times. Most of the sweeteners could form charged molecules in polar mobile phases commonly used in reverse-phase LC systems. The degree of ionization of a molecule will affect interactions between the molecule and the stationary phase. The nine sweeteners were separated most effectively using methanol and 10 mmol/L ammonium acetate solution (at pH 3.8) as the mobile phases and using a gradient elution program. For more information on optimization of pH, mobile phase, and gradient conditions, please see the Supplementary Materials.

### 2.2. Sample Preparation

#### 2.2.1. Sample Preparation with Nitrogen Blowing

**Figure 3:**
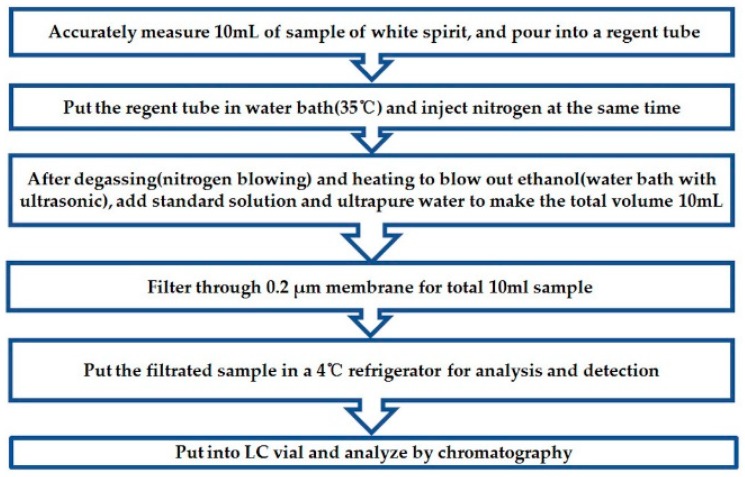
Sample preparation with nitrogen blowing.

The samples were successfully prepared (i.e., the matrices were simplified) by evaporating them under a gentle stream of nitrogen. Due to the large amount of ethanol in liquor, it was easy to affect the retention performance of the sweetener in the process of liquid phase separation. Therefore, it was necessary to dealcoholize the wine samples to be tested in advance. In order to avoid the loss of other components in the sample caused by a temperature increase, we adopted the nitrogen blowing method at 35 °C. Figure 3 shows the workflow of the sample preparation with nitrogen blowing.

We also tried sample pretreatment without nitrogen and solid phase extraction (SPE). The processes of sample pretreatment without nitrogen was basically the same as treatment with nitrogen except the second part. The regent tube was put into the water bath and did not inject nitrogen. For the SPE steps, please see the Supplementary Materials.

**Figure 4:**
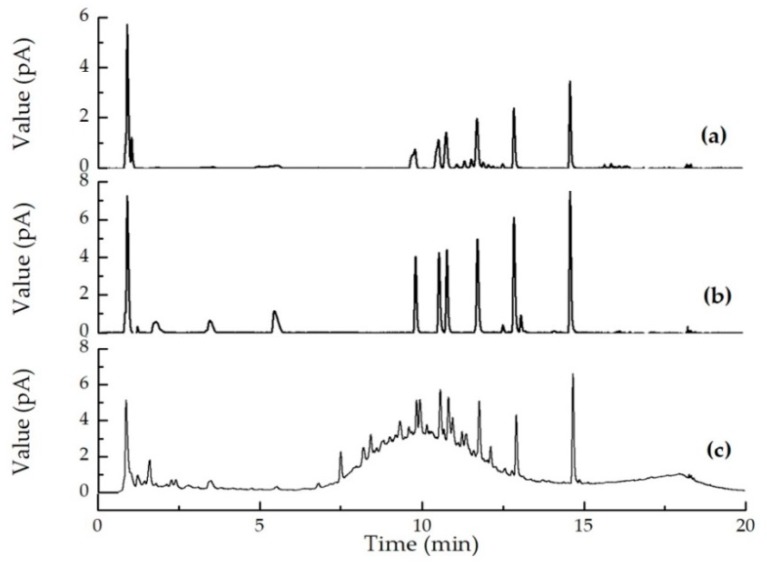
Chromatograms of the pretreatment for spirit samples (white spirit samples added 10 μg/g of sweetener solution and treated respectively (**a**) without the pretreatment of nitrogen, (**b**) evaporation under a gentle stream of nitrogen, and (**c**) solid phase extraction treatment).

Evaporating a sample under a gentle stream of nitrogen was a more effective treatment than solid phase extraction. It took ~25 min to prepare a sample by evaporating it under a gentle stream of nitrogen, and the cost was ~3.0 yuan per sample. Figure 4-(a) shows that ACS-K, SAC, and CYC could hardly be detected, and the chromatographic peak of SCL was seriously deformed. The solid phase extraction procedure took 55 min using a fully automatic solid phase extraction unit, and cost ~8.0 yuan per sample (for the extraction column, solvent, and other materials). Figure 4-(b) (for a sample that was evaporated under a gentle stream of nitrogen) shows a low baseline and good symmetry. Preparing a sample by evaporating it under a gentle stream of nitrogen was simple, fast, and cheap.

#### 2.2.2. Selection of Filter Membrane

**Figure 5:**
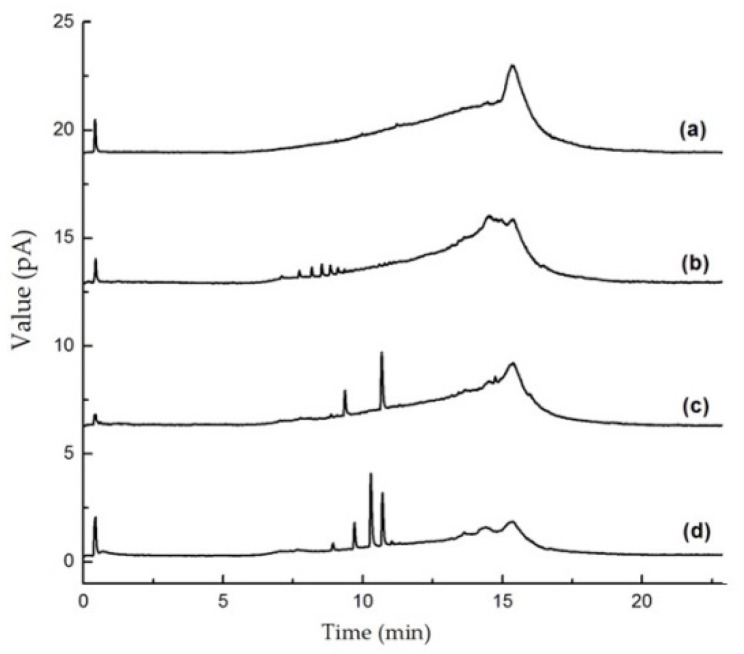
Chromatograms of blank water detected by four kinds of membranes. ((**a**) Polyethersulfone microporous membrane (PES) 0.20 μm, (**b**) Nylon microporous membrane, 0.20 μm, (**c**) polyvinylidene fluoride (PVDF) membrane 0.20 μm, (**d**) Polytetrafluoroethylene membrane (PTFE) 0.20 μm).

We also analyzed the microporous filter membrane, which significantly affected the preparation results. The mixed standard solution used in the experiment needed to be processed by a vortex oscillator before detection to ensure that the sweetener was fully dissolved. However, there were still some particles or impurities in it. If the sample was injected directly, it was easy to block the chromatographic column and shorten the life of the column; at the same time, it also had a certain impact on the detector. Therefore, it was necessary to filter the samples to be tested with a microporous filter membrane to protect the components, such as the column and inject valve, from pollution. The membrane used in the experiment was investigated and analyzed, as shown in Figure 5.

We compared four microporous membranes. The blank water was treated with nylon, PVDF, PTFE, and PES, respectively, then directly injected in UHPLC-CAD. The results showed some impurity peaks after about 10 min of nylon, PVDF, and PTFE. The probable reason was that a small amount of chemical substances on the membrane fell off, which could also be detected. The modified PES microporous membrane had chemical and thermal stability, and acid and alkali resistance (pH 1–14), thus ensuring low dissolution and good reproducibility. Through comparison, the PES microporous membrane was used to treat the mixed standard solution and the real sample.

### 2.3. Method Validation and Application of the Method to Real Samples

#### 2.3.1. UHPLC-PDA-CAD Chromatogram

**Figure 6:**
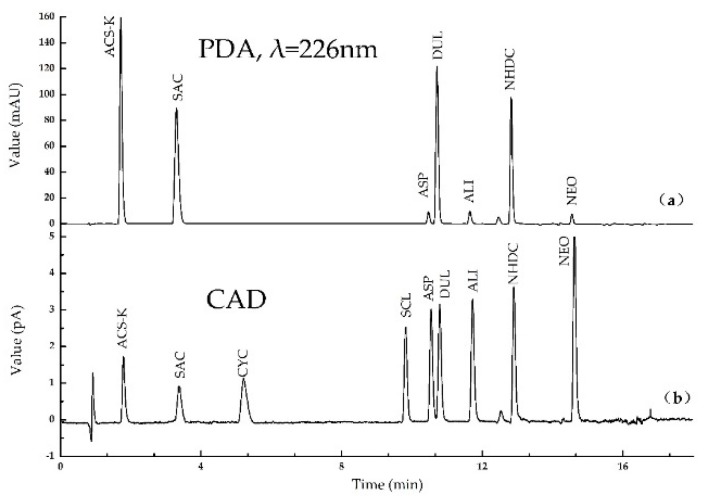
Ultrahigh performance liquid chromatography chromatograms for a standard solution containing each sweetener at a concentration of 10 μg/g acquired using the photo-diode array detector ((**a**), using a wavelength of 226 nm) and using the charged aerosol detector (**b**).

The nine sweeteners were quantified by analyzing calibration standard solutions using the same UHPLC conditions that were used for the white spirits. The nine sweeteners were effectively separated within 16 min using the optimum conditions. Typical UHPLC-PDA-CAD chromatograms for the target compounds are shown in Figure 6. All the analytes were determined simultaneously by UHPLC-PDA-CAD using the rapid column. The chromatograms indicated that the UHPLC resolution and peak shapes were acceptable. Four compounds (including ACS-K, DUL, NHDC, and SAC) were identified in the PDA chromatogram acquired at a wavelength of 226 nm. All the sweeteners were identified in the CAD chromatogram and were able to be quantified.

All 30 products from 12 different brands were collected from different areas in Beijing supermarkets (China). All the samples were stored under refrigeration conditions (4 °C) until analysis. Because all the sweeteners in the present study had good solubility in water, the food samples could be directly used for sweetener analysis. The samples were treated using the nitrogen blow method, adjusted the pH to 3.8, and filtered through a 0.20μm syringe filter prior to being injected into UHPLC-PDA-CAD for analysis.

#### 2.3.2. Linear Ranges, Regression Equations, the Limit of Detection (LOD), the Limit of Quantization (LOQ), Repeatability and Reproducibility

The linear ranges of the calibration curves for the sweeteners were determined. For the CAD data, the calibrations were linear over the concentration range 0.5–50.0μg/g, and the coefficients of determination (*γ*^2^) were 0.9937 to 0.9963. The repeatability was determined by analyzing standard solutions containing the sweeteners, each at a concentration of 5.0μg/g, and the results are shown in Table 1. The repeatability for the sweeteners was 1.2% to 3.1% (CAD) and the reproducibility was 2.3% to 3.6% (CAD). These results indicated that the method was precise and fit for purpose. The LOD and LOQ were defined as the concentrations giving signal-to-noise ratios of 3 and 10, respectively. The LOD and LOQ for the sweeteners are shown in Table 1. See the Supplementary Materials for the linearity with PDA and CAD (Supplementary Materials S3, S4).

The LOD for the CAD data were 0.36 μg/g for ACS-K, 0.19 μg/g for ALI, 0.20 μg/g for ASP, 0.32 μg/g for CYC, 0.18 μg/g for DUL, 0.16 μg/g for NEO, 0.16 μg/g for NHDC, 0.77 μg/g for SAC, and 0.18 μg/g for SCL. The LOD for the PDA data were 0.16 μg/g for ACS-K, 0.18 μg/g for DUL, 0.21 μg/g for NHDC, and 0.22 μg/g for SAC. These LOD were much lower than those found in previous studies [9,19,20,21,22,31] for methods involving HPLC evaporative light-scattering detection and HPLC-MS (13.0 μg/g for ACS-K, 2.0 μg/g for ALI, 10.0 μg/g for ASP, 1.0 μg/g for CYC, 6.0 μg/g for DUL, 5.0 μg/g for NEO, 2.0 μg/g for NHDC, 2.0 μg/g for SAC, and 1.0 μg/g for SCL). This clearly indicates that the method described here was very effective for analyzing sweeteners in white spirits. This was particularly the case for ALI, NHDC, and SCL, which had LOD much lower than required. Table 2 compares the LOD and LOQ in the related literature.

#### 2.3.3. Recoveries and Accuracy

The accuracy of the method was assessed by analyzing three white spirit samples. The samples contained 38°, 46°, and 52° white spirits (calculated with ethanol). Each sample was spiked with the nine sweeteners at fortification levels (for each sweetener) of 5.0, 20.0, and 40.0 μg/g. The recoveries are presented in Table 3. Good recoveries were obtained for the 38° spirit samples (95.9–104.5% recoveries), the 46° spirit samples (95.1–103.8% recoveries), and the 52° spirit samples (95.5–104.9% recoveries). As shown in Table 3, the precisions were 2.5% to 3.8% for the 38° spirit samples, 2.2% to 3.4% for the 46° spirit samples, and 1.6% to 3.0% for the 52° spirit samples.

### 2.4. Real Sample Analysis

**Figure 7:**
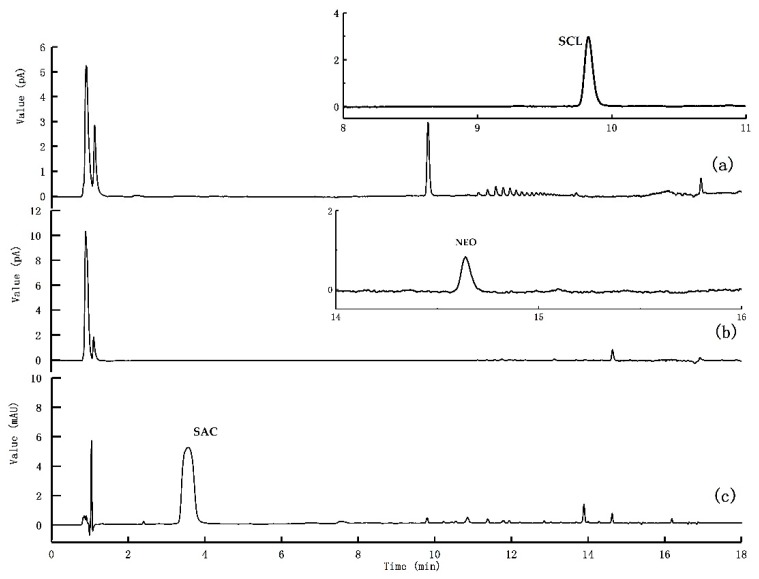
Ultrahigh performance liquid chromatography photo-diode array detector and charged aerosol detector chromatograms for samples that were found to contain sweeteners.

The concentrations of the nine sweeteners in 30 real white spirit samples (12 brands), containing either 38°, 46°, or 52° alcohol were determined (Supplementary Materials Table S1). Three samples were found to contain sweeteners. Sample 1 (a) contained SCL at a concentration of 8.45μg/g (determined using the CAD data). Sample 7 (b) contained NEO at a concentration of 1.07μg/g (determined using the CAD data). Sample 12 (c) contained SAC at a concentration of 3.22μg/g (determined using the PDA data). The chromatograms for these samples are shown in Figure 7. The LOD allowed the low concentrations of the sweeteners in the white spirit samples to be determined. The artificial sweetener content contained in sample No.1 was outside of the legal limit according to GB 2760.

## 3. Materials and Methods

### 3.1. Instrumentation and Reagents

Chromatograms were acquired using a UHPLC system consisting of two GP40 LC pumps, an AS50 autosampler, an LC 20 column compartment, an Ultimate 3000 PDA detector, and a Corona Ultra CAD, with the detectors linked using a series connection (Thermo Fisher Scientific, Waltham, MA, USA). Nine food sweetener reference materials were provided by the National Institute for Metrology of China (Beijing, China). The sweetener reference materials (RM) were ACS-K (GBW(E)100065, 99.6% ± 0.6% pure), ALI (98.3% ± 0.7% pure), ASP (99.0% ± 0.7% pure), CYC (GBW(E)100066, 99.3% ± 0.7% pure), DUL (98.0% ± 1.0% pure), NEO (98.7% ± 0.6% pure), NHDC (99.0% ± 0.6% pure), SAC (GBW10006, 99.5% ± 0.5% pure), and SCL (GBW(E)100132, 99.6% ± 0.5% pure). HPLC-grade ammonium acetate and formic acid were purchased from Sigma-Aldrich (St. Louis, MO, USA). Methanol and acetonitrile (HPLC grade) were purchased from Merck (Darmstadt, Germany). Further, 0.20μm, polyethersulfone(PES), nylon, polyvinylidene fluoride (PVDF) and polytetrafluoroethylene(PTFE) four membranes (Shanghai ANPEL Scientific Instrument Co., Ltd.) adsorbents were provided by Agela Technologies company (Tianjin, China). Automatic solid phase extractor, Fotector-06C, Reeko (Xiamen, China). Solid phase extraction column, Strata-X 33μm (3 mL/200 mg), Chromabond C18 (6 mL/1000 mg), Agela Technologies company (Tianjin, China).

### 3.2. Preparation of Standard Solutions and Samples

The standard solutions were prepared on a weight–weight basis. ACS-K, CYC, SAC, and SCL solutions were each prepared by dissolving an aliquot of the relevant sweetener in deionized water to give a solution containing the sweetener at a concentration of 500 μg/g. ALI, ASP, and NEO solutions were each prepared by dissolving an aliquot of the relevant sweetener in 10 mmol/L ammonium acetate solution to give a solution containing the sweetener at a concentration of 500 μg/g. DUL and NHDC solutions were each prepared by dissolving an aliquot of the relevant sweetener in deionized water to give a solution containing the sweetener at a concentration of 100 μg/g. Working mixed standard solutions at concentrations lower than 1.0 μg/g were prepared by diluting 1.0 μg/g mixed standard solutions as required. Standard solutions of the sweeteners at concentrations between 0.5 and 50.0 μg/g were prepared by performing serial dilutions starting with high concentration standard solutions. In total, 12 brand real white spirits for 30 samples, were obtained from local markets.

### 3.3. UHPLC-PDA-CAD Conditions

Separation of the sweeteners was achieved using an Acquity UHPLC BEH C18 column (2.1 mm i.d., 100 mm long, 1.7-μm particle size, Waters Corporation, made in Ireland) using a gradient profile. The mobile phases were (A) methanol and (B) 10 mmol/L ammonium acetate solution, and the gradient elution program started at 10% A, which was held between 0 and 3 min, increased linearly to 90% A between 3 and 13 min, remained at 90% A until 15 min, and then returned to 10% A. The mobile phase flow rate was 0.3 mL/min. The column oven temperature was 35 °C. The injection volume was 5 μL. The PDA detector monitoring wavelength range was 190–700 nm, The CAD detector nebulizer temperature was 35 °C, the gas pressure was 0.24 MPa, and the data collection rate was 20 Hz.

### 3.4. Method Validation

Quantitative analysis was performed using an external standards calibration method. The calibration solutions were prepared by diluting intermediate mixed aqueous standard solutions to give sweetener concentrations between 0.5 and 50.0 μg/g. The LOD and LOQ were defined as the concentrations giving signal-to-noise ratios of 3 and 10, respectively. Repeatability (intra-day precision) was assessed by analyzing a standard solution containing the sweeteners each at a concentration of 5.0 μg/g seven times in one day. Reproducibility (inter-day precision) was assessed by two different analysts analyzing the same standard twice each day for five days. Accuracy was assessed by performing recovery experiments using three wine samples with different origins spiked with the nine sweeteners at concentrations of 5.0, 10.0, and 40.0 μg/g. Each spiked sample was analyzed seven times. Matrix effects were assessed by adding standard sweetener solutions to a blank white spirit sample to give final sweetener concentrations of 5.0 and 20.0 μg/g and then analyzing the samples.

## 4. Conclusions

An efficient and accurate method was developed for simultaneously determining nine sweeteners in Chinese spirits by UHPLC-PDA-CAD. The method included a simple sample preparation procedure (evaporation under a gentle stream of nitrogen and filtration), and is sensitive, cheap, simple, and quick. The method allows synthetic and semi-synthetic high-intensity sweeteners to be detected at low concentrations (micrograms per gram). The applicability of the method was verified by determining sweeteners in 30 real spirit samples. Finally, the method was successfully used in a major project called “Special Action against Counterfeit and Shoddy white spirits” and to monitor risks posed by white spirits in China.

## Figures and Tables

**Table 1:** Chromatographic data, linear ranges, regression equations ^a^, correlation coefficients, limits of detection ^b^, limits of quantitation ^c^, repeatability ^d^, and reproducibility ^e^ for the nine sweeteners in white spirits using the ultrahigh performance liquid chromatograph photo-diode array detector and charged aerosol detector method.

Analytes	t_R_ ± S (min)			CAD						PDA (λ=226nm)					
		Resolution	Linear Ranges ^f^	Linear Equation	*γ^2^*	LOD	LOQ	Repeatability	Reproducibility	Linear Equation	*γ^2^*	LOD	LOQ	Repeatability	Reproducibility
**ACS-K**	1.79 ± 0.09	8.67	1.0–50.0	y = 0.0124 x + 0.0212	0.9946	0.36	1.06	2.4%	3.1%	y = 1.275 *x* − 0.124	0.9998	0.16	0.50	1.1%	1.2%
**SAC**	3.35 ± 0.02	6.89	2.0–50.0	y = 0.0109 x + 0.0067	0.9937	0.77	2.07	2.1%	3.0%	y = 1.183 *x* − 0.188	0.9999	0.22	0.60	0.9%	1.3%
**CYC**	5.19 ± 0.03	19.9	1.0–50.0	y = 0.0207 x + 0.0318	0.9963	0.32	0.95	3.1%	3.6%	/ ^g^	/	/	/	/	/
**SCL**	9.82 ± 0.03	4.68	0.7–50.0	y = 0.0202 x + 0.0446	0.9949	0.18	0.52	2.0%	2.3%	/	/	/	/	/	/
**ASP**	10.55 ± 0.03	1.58	0.7-50.0	y = 0.0237 x + 0.0523	0.9942	0.20	0.59	1.2%	2.9%	/	/	/	/	/	/
**DUL**	10.80 ± 0.04	5.84	0.5-50.0	y = 0.0265 x + 0.0524	0.9956	0.18	0.54	2.0%	3.4%	y = 1.056 *x* − 0.050	0.9999	0.18	0.50	0.8%	1.6%
**ALI**	11.72 ± 0.03	7.64	0.5–50.0	y = 0.0285 x + 0.0531	0.9957	0.19	0.59	1.4%	3.1%	/	/	/	/	/	/
**NHDC**	12.91 ± 0.07	11.9	0.5–50.0	y = 0.0313 x + 0.0427	0.9963	0.16	0.53	1.6%	2.9%	y = 0.771 *x* − 0.288	0.9993	0.21	1.01	1.0%	1.7%
**NEO**	14.64 ± 0.06	3.51	0.5–50.0	y = 0.0425 x + 0.0714	0.9953	0.16	0.49	1.5%	3.0%	/	/	/	/	/	/

Notes: ^a^ calibration curves with different compounds at concentrations of 0.5, 1.0, 2.0, 5.0, 10.0, 20.0, and 50.0 μg/g. ^b^ the limit of detection (LOD) was evaluated based on a signal-to-noise ratio of 3 (*S/N*), unit: μg/g. ^c^ the limit of quantization (LOQ) was evaluated based on a signal-to-noise ratio of 10 (*S/N*), unit: μg/g. ^d^ the repeatability (n = 7). Compound concentration at 5.0 μg/g. ^e^ the reproducibility (n = 5, two analysts, 2 times/day). Compound concentration at 5.0 μg/g. ^f^ the liner range unit: μg/g. ^g^ not available, as CYC, SCL, ASP, ALI, NEO did not respond at 226 nm (PDA).

**Table 2:** Comparison of the limit of detection(LOD) and limit of quantization(LOQ) in the related literatures.

	Matrices	Analytes	LOD	LOQ	Ref
UPLC-UV	juices	ACS-K	0.75 μg/mL	NA	[9]
		ASP	0.75 μg/mL		
		SAC	0.30 μg/mL		
HPLC-ELSD	canned fruits, yoghurt, energy drink	ACS-K	13.0 μg/g	NA	[19]
		ALI	2.0 μg/g		
		ASP	10.0 μg/g		
		CYC	1.0 μg/g		
		DUL	6.0 μg/g		
		NHDC	2.0 μg/g		
		NEO	5.0 μg/g		
		SAC	2.0 μg/g		
		SCL	1.0 μg/g		
HPLC-ELSD	commercial samples	SCL	0.5 μg/mL	2.0 μg/mL	[20]
LCMS	food	ACS-K	NA	1–5 μg/g	[21]
		SCL			
		SAC			
		CYC			
		ASP			
		DUL			
LCMS (ion-pair)	food	CYC	1 ng/mL	5 ng/mL	[22]
HPLC-CAD-UV/DAD	soft drinks	ASP	0.08–0.20 μg/mL	0.19–0.61 μg/mL	[32]
		ACS-K			
		SAC			
UHPLC-PDA-CAD	white spirits	ACS-K	0.36 μg/g	1.06 μg/g	Present method
		ALI	0.19 μg/g	0.59 μg/g	
		ASP	0.20 μg/g	0.59 μg/g	
		CYC	0.32 μg/g	0.95 μg/g	
		DUL	0.18 μg/g	0.54 μg/g	
		NHDC	0.16 μg/g	0.53 μg/g	
		NEO	0.16 μg/g	0.49 μg/g	
		SAC	0.77 μg/g	2.07 μg/g	
		SCL	0.18 μg/g	0.52 μg/g	

**Table 3:** Recovery and accuracy results for determining nine sweeteners in three different spirit samples.

Analytes	Added (μg/g)	White Spirits 38°			White Spirits 46°			White Spirits 52°		
		Found	Recovery	RSD	Found	Recovery	RSD	Found	Recovery	RSD
ACS-K	5.0	4.88	97.6%	3.0%	4.74	94.8%	2.8%	4.80	96.0%	2.5%
	10.0	9.59	95.9%	3.2%	9.67	96.7%	2.7%	9.71	97.1%	2.2%
	40.0	38.49	96.2%	2.9%	38.18	95.4%	2.5%	39.04	97.6%	2.1%
CYC	5.0	5.23	104.5%	3.6%	5.12	102.5%	3.4%	5.25	104.9%	2.9%
	10.0	10.20	102.0%	3.8%	10.28	102.8%	3.2%	10.21	102.1%	3.0%
	40.0	40.32	100.8%	3.1%	41.15	102.9%	2.9%	41.56	103.9%	2.6%
SAC	5.0	4.80	96.0%	3.1%	4.76	95.1%	2.8%	4.78	95.5%	2.3%
	10.0	9.63	96.3%	3.0%	9.78	97.8%	2.6%	9.69	96.9%	2.2%
	40.0	39.64	99.1%	3.3%	40.33	100.8%	3.0%	40.25	100.6%	2.3%
SCL	5.0	4.99	99.8%	2.9%	5.04	100.8%	2.2%	4.98	99.5%	2.3%
	10.0	9.96	99.6%	2.7%	10.02	100.2%	2.8%	9.95	99.5%	2.6%
	40.0	40.33	100.8%	3.1%	39.88	99.7%	2.7%	39.67	99.2%	2.4%
ASP	5.0	4.98	99.7%	2.8%	5.13	102.6%	2.3%	4.90	98.1%	1.6%
	10.0	9.97	99.7%	2.5%	9.81	98.1%	2.4%	9.89	98.9%	1.9%
	40.0	39.00	97.5%	2.7%	38.62	96.5%	2.4%	39.24	98.1%	2.0%
DUL	5.0	4.86	97.1%	3.3%	4.72	94.3%	2.3%	4.82	96.3%	2.7%
	10.0	10.01	100.1%	3.0%	9.82	98.2%	2.9%	9.62	96.2%	2.6%
	40.0	38.56	98.4%	3.2%	38.80	97.0%	2.8%	39.93	99.8%	2.4%
ALI	5.0	4.79	98.8%	3.3%	4.76	98.2%	3.0%	4.76	97.2%	2.4%
	10.0	9.90	96.0%	3.1%	9.97	97.7%	2.6%	9.77	97.7%	1.9%
	40.0	38.76	97.0%	3.4%	39.07	97.7%	2.9%	38.88	97.3%	2.3%
NHDC	5.0	4.89	97.8%	3.1%	4.81	96.2%	2.2%	4.90	98.1%	1.9%
	10.0	9.88	98.8%	3.0%	9.97	99.7%	2.5%	9.93	99.3%	2.2%
	40.0	39.39	98.5%	3.3%	39.01	97.5%	2.6%	39.08	97.7%	2.3%
NEO	5.0	4.91	98.2%	2.9%	4.79	95.9%	2.6%	4.96	99.1%	1.8%
	10.0	9.69	96.9%	3.3%	9.81	98.1%	2.7%	9.82	98.2%	2.1%
	40.0	38.51	96.3%	3.0%	38.74	96.9%	2.8%	38.71	96.8%	2.3%

## Supplementary Materials

The following are available online at https://www.mdpi.com/1420-3049/25/1/40/s1, Figure S1: Area differentiation of nine sweeteners adjusting pH with different concentration of ammonium acetate, Figure S2: The effect of different mobile flows on separation, Figure S3: Linearity of nine sweeteners(PDA), Figure S4: Linearity of nine sweeteners (CAD), Figure S5: Optimization of pretreatment conditions, Figure S6: Workflows of three preparation methods, Table S1: Three of the thirty samples detected illegally added sweetener, Table S2: Operation order list of solid phase extraction, Table S3: The Resolution and Tailing factor of nine sweeteners by UHPLC-CAD, Table S4: The separation gradient condition for nine sweeteners.
