# Supplementary material for: Determining High-Intensity Sweeteners in White Spirits Using an Ultrahigh Performance Liquid Chromatograph with a Photo-Diode Array Detector and Charged Aerosol Detector
Source: Molecules. 2019 Dec 20;25(1):40. doi: 10.3390/molecules25010040 (PMC6983009; doi:10.3390/molecules25010040)
Supplement: Supplementary file 1 [file molecules-25-00040-s001.pdf]

molecules

MDPI

Supplement Materials

1). Figure S1 Area differentiation of nine sweeteners adjusting pH with different concentration of ammonium acetate

2). Figure S2 The effect of different mobile flows on separation (a) Methanol Versus (b)Acetonitrile

3). Figure S3 Linearity of nine sweeteners (PDA, λ=226nm)

4). Figure S4 Linearity of nine sweeteners (CAD)

5). Figure S5 Optimization of pretreatment conditions (with and without nitrogen blowing)

6). Figure S6 Workflows of three preparation methods

7). Table S1 Three of the thirty samples detected illegally added sweetener

8). Table S2 Operation order list of solid phase extraction

9). Table S3 The Resolution and Tailing factor of nine sweeteners by UHPLC-CAD

10). Table S4 The separation gradient condition for nine sweeteners

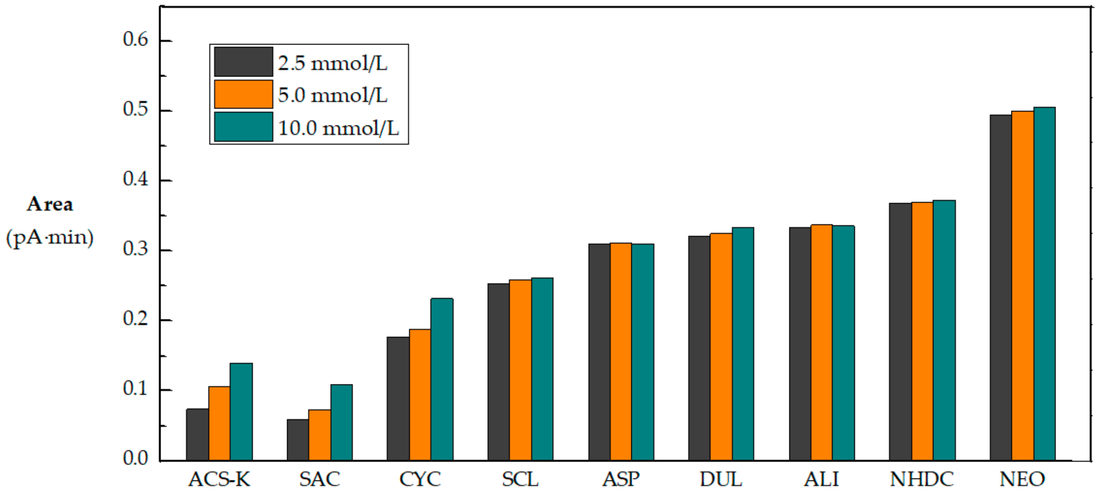

Figure S1. Area differentiation of nine sweeteners adjusting pH with different concentration of ammonium acetate.

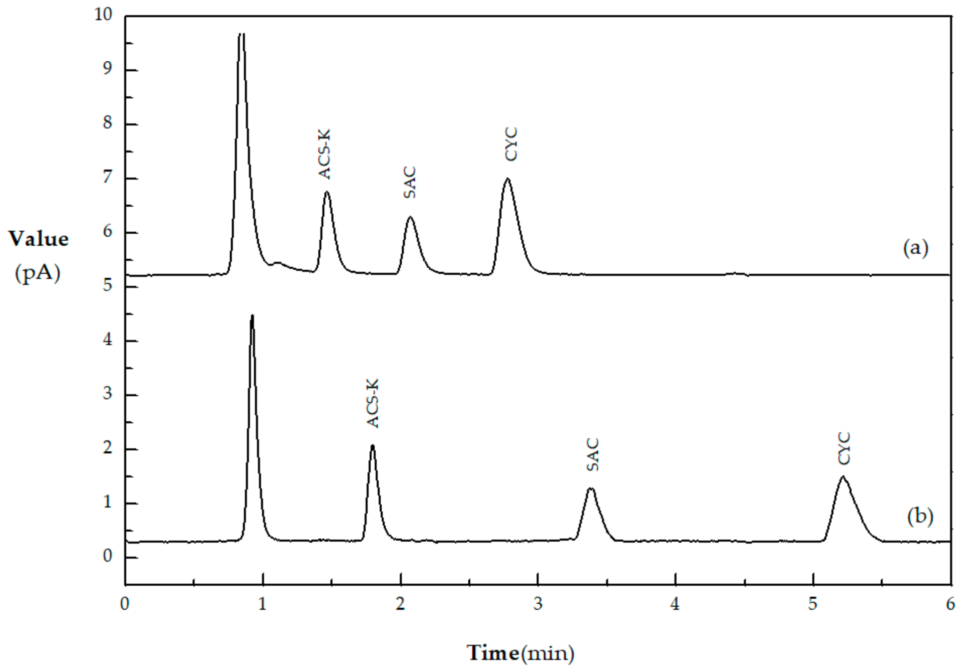

Figure S2. The effect of different mobile flows on separation (a) Acetonitrile Versus (b) Methanol.

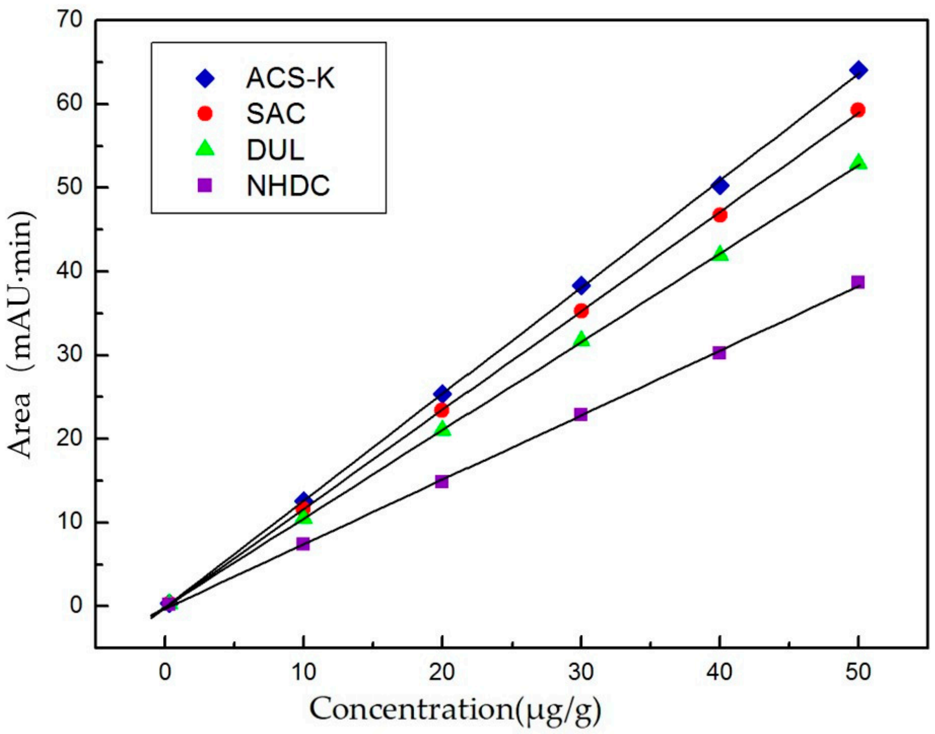

Figure S3. Linearity of nine sweeteners (PDA, λ=226nm).

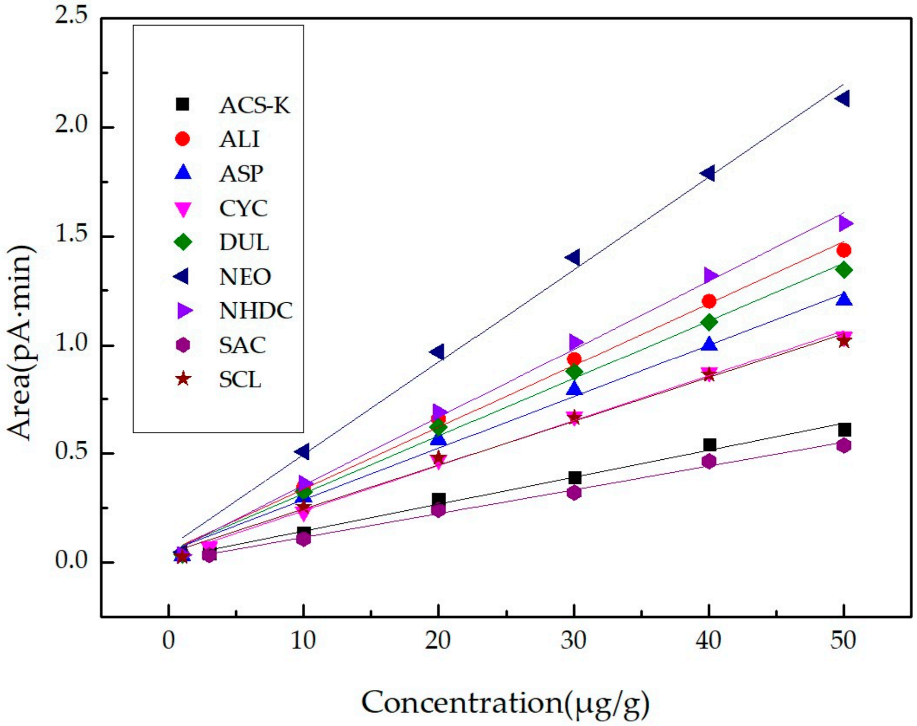

Figure S4. Linearity of nine sweeteners (CAD).

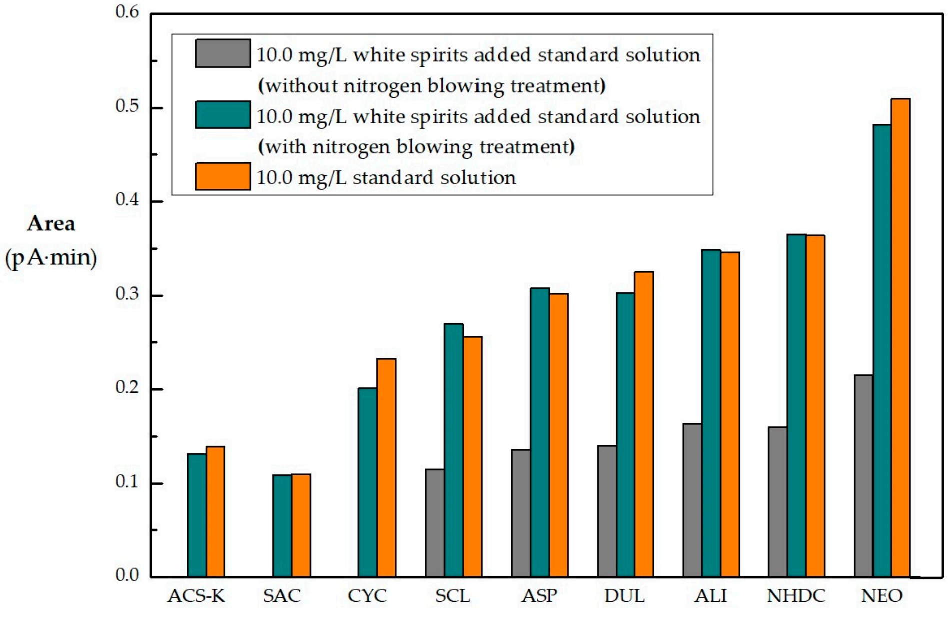

Figure S5. Optimization of pretreatment conditions (with and without nitrogen blowing).

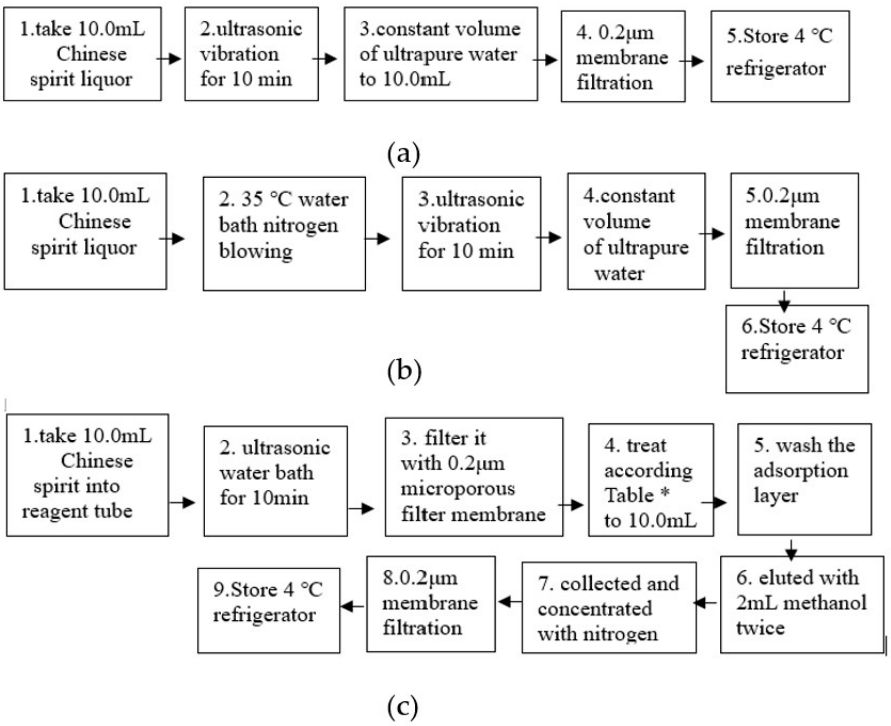

Figure S6. Workflows of three preparation methods. (a)sample preparation without nitrogen blowing (b)sample preparation with nitrogen blowing (c) sample preparation with SPE (Table* see supplementary Table 2).

Table S1. Three of the thirty samples detected illegally added sweetener.

CAD DETECTOR									
Sample(μg/g)	ACS-K	ALI	ASP	CYC	DUL	NEO	NHDC	SAC	SCL
1(46°)	ND*	ND*	ND*	ND*	ND*	ND*	ND*	ND*	8.45
7(38°)	ND*	ND*	ND*	ND*	ND*	1.07	ND*	ND*	ND*
**PDA DETECTOR**									
Sample(μg/g)	ACS-K	ALI	ASP	CYC	DUL	NEO	NHDC	SAC	SCL
12(38°)	ND*	NA**	NA**	NA**	ND*	NA**	ND*	6.22	NA**

*ND Not detected, less than detection limit (LOD), **NA Not available.

Table S2. Operation order list of solid phase extraction.

Operation order	solvent	flow velocity /(mL/min)	volume /mL	time/min
clean the injection pump	methanol	3	5	2.17
activation	methanol	2	3	1.80
clean the injection pump	buffer solution	3	5	2.17
activation	buffer solution	2	6	3.60
pneumatic push	/	10	10	1.00
suspend	/	/	/	1.00
upper sample	/	1	5	5.50
Clean sample bottle	buffer solution	100	5	6.05
elution	buffer solution	1.5	5	3.83
pneumatic push	/	10	10	1.00
blow dry	/	/	/	10.00
elution	methanol	1.5	2	1.53
suspend	/	/	/	10.00
elution	methanol	1.5	2	1.53
pneumatic push	/	10	10	1.00
concentrate	/	/	/	20.00
end	/	/	/	72.18

Table S3. The Resolution and Tailing factor of nine sweeteners by UHPLC-CAD.

Sweeten-er	Shim-pack		Zorbax		BEH 50 mm		BEH 100 mm	
	Tailing factor	Resolu-tion	Tailing factor	Resolu-tion	Tailing factor	Resolu-tion	Tailing factor	Resolu-tion
ACS-K	1.56	12.8	1.51	6.73	1.50	5.44	1.42	8.67
SAC	1.65	6.51	1.68	4.65	1.39	4.48	1.26	6.89
CYC	1.36	7.25	1.62	21.4	1.40	32.5	1.35	19.9
SCL	1.30	3.10	1.74	4.53	1.15	6.37	1.18	4.68
ASP	/*	/	2.47	2.94	/	/	1.21	1.58
DUL	/	/	1.76	4.08	/	/	1.15	5.84
ALI	/	/	1.74	4.58	1.28	17.2	1.25	7.64
NHDC	/	/	2.49	12.9	1.13	16.0	1.24	11.9
NEO	1.26	n.a.**	1.99	n.a.	1.20	n.a.	1.29	n.a.

* cannot calculated

** not available.

Notes, Shim-pack, Zorbax, BEH 50 mm at the same gradient condition 1 (Figure 2 a,b,c in main manuscript), BEH 100 mm changed gradient condition 2 (Figure 2 d in main manuscript).

Table S4. The separation gradient condition for nine sweeteners.

separation gradient condition 1			separation gradient condition 2		
Time	A%	B%	Time	A%	B%
0.000	10.0	90.0	0.000	10.0	90.0
3.000	10.0	90.0	3.000	10.0	90.0
10.000	85.0	15.0	13.000	90.0	10.0
15.000	80.0	20.0	15.000	90.0	10.0
15.010	10.0	90.0	15.010	10.0	90.0
25.000	10.0	90.0	25.000	10.0	90.0

© 2019 by the authors. Licensee MDPI, Basel, Switzerland. This article is an open access article distributed under the terms and conditions of the Creative Commons Attribution (CC BY) license (http://creativecommons.org/licenses/by/4.0/).
